# MorphDB: Prioritizing Genes for Specialized Metabolism Pathways and Gene Ontology Categories in Plants

**DOI:** 10.3389/fpls.2018.00352

**Published:** 2018-03-19

**Authors:** Arthur Zwaenepoel, Tim Diels, David Amar, Thomas Van Parys, Ron Shamir, Yves Van de Peer, Oren Tzfadia

**Affiliations:** ^1^Department of Plant Biotechnology and Bioinformatics, Ghent University, Ghent, Belgium; ^2^VIB Center for Plant Systems Biology, Ghent, Belgium; ^3^Bioinformatics Institute Ghent, Ghent University, Ghent, Belgium; ^4^Stanford Center for Inherited Cardiovascular Disease, Stanford University, Stanford, CA, United States; ^5^Blavatnik School of Computer Science, Tel-Aviv University, Tel-Aviv, Israel; ^6^Genomics Research Institute, University of Pretoria, Pretoria, South Africa

**Keywords:** comparative co-expression networks, candidate gene prioritization, functional annotation, MORPH, defense response

## Abstract

Recent times have seen an enormous growth of “omics” data, of which high-throughput gene expression data are arguably the most important from a functional perspective. Despite huge improvements in computational techniques for the functional classification of gene sequences, common similarity-based methods often fall short of providing full and reliable functional information. Recently, the combination of comparative genomics with approaches in functional genomics has received considerable interest for gene function analysis, leveraging both gene expression based guilt-by-association methods and annotation efforts in closely related model organisms. Besides the identification of missing genes in pathways, these methods also typically enable the discovery of biological regulators (i.e., transcription factors or signaling genes). A previously built guilt-by-association method is MORPH, which was proven to be an efficient algorithm that performs particularly well in identifying and prioritizing missing genes in plant metabolic pathways. Here, we present MorphDB, a resource where MORPH-based candidate genes for large-scale functional annotations (Gene Ontology, MapMan bins) are integrated across multiple plant species. Besides a gene centric query utility, we present a comparative network approach that enables researchers to efficiently browse MORPH predictions across functional gene sets and species, facilitating efficient gene discovery and candidate gene prioritization. MorphDB is available at http://bioinformatics.psb.ugent.be/webtools/morphdb/morphDB/index/. We also provide a toolkit, named “MORPH bulk” (https://github.com/arzwa/morph-bulk), for running MORPH in bulk mode on novel data sets, enabling researchers to apply MORPH to their own species of interest.

## Introduction

Groups of genes involved in a common biological process are often defined as pathways, which are traditionally studied as if they were isolated groups. However, pathway boundaries are inherently fuzzy which greatly compromises their systematic delineation. In plants, the understanding of secondary metabolism and stress regulated pathways is of paramount importance and even though these pathways have been studied extensively, discovering missing genes and understanding the regulatory interrelations among them remains a fundamental challenge. Moreover, despite more than two decades of functional genomics research, the functions of most plant genes remain unknown. These problems are exacerbated in newly sequenced genomes and non-model organisms (Rhee and Mutwil, 2014). Sequence similarity based tools such as Blast2GO (Conesa et al., 2005; Conesa and Götz, 2008), BlastKOALA (Kanehisa et al., 2016), PlantCyc's EP2P (Schläpfer et al., 2017) and InterProScan (Zdobnov and Apweiler, 2001; Jones et al., 2014), are often used to provide a first clue about the function of a gene in a newly sequenced and annotated genome. Other comparative genomics methods aim to leverage annotation efforts in model organisms, typically by utilizing clustering analysis, using e.g., OrthoMCL (Li et al., 2003) or OrthoFinder (Emms and Kelly, 2015). After the delineation of groups of homologous genes, annotations are transferred between orthologs under the assumption that evolutionary conservation implies a conserved function.

A complementary approach for gene function prediction is to use “omics” data (e.g., transcriptomics and proteomics) within an integrative analysis pipeline that builds on the guilt-by-association (GBA) principle. GBA involves inferring putative gene functions for unknown genes from genes with known functions that show similar behavior across different experimental conditions or data sets. For example, co-expression based GBA with genes from known Gene Ontology (GO) terms has been shown to be ubiquitously applicable across the transcriptome of different species (Wolfe et al., 2005). Because of the demonstrated general applicability of the GBA principle and the fact that transcriptomic data is the most straightforward 'omics' data to gather, there is an increasing usage of co-expression networks for candidate gene prioritization in the plant science community (Rhee and Mutwil, 2014; Serin et al., 2016).

A related methodology for in-depth analysis of gene functions is comparative transcriptomics, in which evolutionary relationships between genes are used to integrate expression data across species (Movahedi et al., 2011, 2012; Hansen et al., 2014). Such methods often use integrative network approaches to allow discovery of conserved co-expression modules (Zarrineh et al., 2011) across multiple species, again possibly leveraging knowledge from model to non-model organisms. These networks can often unveil missing pathway genes and regulators, as they naturally cope with the fuzzy nature of pathway boundaries while incorporating evolutionary relationships that can serve as constraints and can discriminate between highly interesting evolutionary conserved candidate genes and potential noise. Indeed, it has been shown that comparative co-expression networks may yield more accurate gene function predictions (Hansen et al., 2014). Some important (comparative) co-expression based tools for gene function analysis are ATTED-II (Aoki et al., 2016), PlaNet (Proost and Mutwil, 2017), CORNET (De Bodt et al., 2010; Van Bel and Coppens, 2017), AraNet (Lee et al., 2015), MORPH (Tzfadia et al., 2012; Amar et al., 2015), and CoExpNetViz (Tzfadia et al., 2016).

While co-expression based methods are extremely relevant for gene function analysis, some important caveats are to be noted. First and foremost, these networks are based on correlation measures which are prone to spurious associations, indirect functional links, and noise (both false positives and false negatives) (Mutwil et al., 2011; Hansen et al., 2014). Therefore, when analyzing large data sets, co-expression networks quickly become dense, limiting their biological interpretability (Usadel et al., 2009; Serin et al., 2016). Second, and associated with these issues, is the problem of reproducibility, as many distinct steps and filtering decisions have to be taken to produce a co-expression network, while a standardized protocol does not exist. Third, these networks are more suitable for inference of biological processes than of molecular functions (Hansen et al., 2014). Fourth, the conditions, tissues and perturbations used in the expression compendium are also of great importance, especially when one is interested in a specific tissue or condition-dependent regulatory processes (Hansen et al., 2014; Serin et al., 2016). Finally, co-expression analysis is expected to be more suitable for genes and processes under strong transcriptional control, whereas they are not well-suited for processes that are mostly controlled at the translational or post-translational level. For example, Kleessen et al. (2013) showed that co-expression based GBA performs much better for primary and secondary metabolism pathways than for hormone and cell wall related biological processes. These reasons also make it desirable to have some estimate of the performance of GBA on a particular process of interest. A distinct and more practical issue is that most available tools (see above) cannot be easily applied to custom data sets or novel species, limiting their usage to a handful of model organisms.

MORPH (MOdule-guided Ranking of candidate PatHway genes) is an algorithm for unveiling missing genes in biological pathways (Tzfadia et al., 2012; Amar et al., 2015) and uses multiple expression datasets and clustering thereof for the prioritization of candidate genes. As with other gene expression based GBA methods, it relies on an input set of 'bait' genes that are associated with the biological process of interest, and uses the expression profiles of these bait genes across conditions to prioritize candidate genes. MORPH uses clustering solutions of the expression data to calculate a module partitioned co-expression metric for each candidate gene with regard to the input bait genes. Based on the input set of bait genes, MORPH selects the optimal expression data—clustering combination to be used for the prioritization of candidate genes. This configuration learning step follows an approach commonly known in machine learning as “model selection” (Guyon et al., 2010). To this end, MORPH uses a leave-one-out cross validation (LOOCV) procedure. For every gene *g*_*i*_ in a given bait gene set *G*, the MORPH algorithm is run with as input bait genes the set *G*′ defined as the set *G* with *g*_*i*_ left out (i.e., *G*′ = *G* \ {*g*_*i*_}). Using this bait gene set, the “self-rank” for *g*_*i*_ is determined, defined as the rank assigned to *g*_*i*_ by MORPH using the set *G*′. The self-ranks for every *g*_*i*_ are collected and can then be plotted in a self-rank curve, which shows for increasing rank threshold, the proportion of bait genes with a rank higher than the threshold. The area under the self-rank curve (AUSR) can then be used as a model selection metric, as the data set—clustering combination that results in the highest AUSR can be regarded as the one most appropriate to use for GBA based candidate gene prioritization. Interestingly, besides its use for model selection, this AUSR metric can also be used as an estimate of the performance (and relevance) of GBA based methods on a process of interest. While powerful and with proven success, GBA and co-expression based methods in general have not been fully exploited and their real value for plant functional genomics is yet to be explored (Rhee and Mutwil, 2014).

In this paper we extend and improve MORPH. We present a new tool, called MorphDB, which covers more organisms and functional annotations, and provides advanced visualizations that can help researchers in performing genome-wide comparative analyses for a series of model organisms. The new analyses (genome-wide and comparative modes, functional annotation of newly sequenced species) are explored using multiple datasets and functional annotations. Gene-centric and process-centric networks are used for visualization of predicted candidate genes across species and functional categories, which is instrumental in guiding knowledge discovery. Several examples of use cases are shown, illustrating the potential of MorphDB for gene discovery and advancing our understanding of plant gene functions. The tool and the results are accessible via a web interface: http://bioinformatics.psb.ugent.be/webtools/morphdb/morphDB/index/. Besides, we offer a framework for running genome-wide MORPH analyses, called “MORPH bulk” (https://github.com/arzwa/morph-bulk), enabling researchers to perform large scale MORPH analyses on their species and data sets of interest.

## Materials and methods

### Expression data processing and functional annotation data

The functional annotation data for the model species was retrieved from the PLAZA 3.0 comparative genomics platform (Van Bel et al., 2012). For *C. roseus* and *Z. marina*, GO annotations were acquired using InterProScan + InterPro2GO and Blast2GO. The expression data and clustering solutions used for *A. thaliana, S. lycopersicum, S. tuberosum, O. sativa*, and *C. roseus* were those already configured for the MORPH web tool (Amar et al., 2015). Expression data for *M. truncatula* was obtained from the *Medicago truncatula* gene expression atlas (Benedito et al., 2008). For *P. trichocarpa* expression data from Shi et al. (2017) was used (GEO accession ID: GSE81077), acquired as count tables. For *Z. marina*, RNA-Seq data from the original genome project was used (Olsen et al., 2016), obtained as both count and fragments per kilobase of exon per million reads mapped (fpkm) data sets (GEO: GSE67579). All expression data sets were filtered by gene-wise standard deviation, such that ~75% of the genes were retained. All microarray data sets were normalized using quantile normalization (Irizarry et al., 2003), while all RNA-Seq data sets were normalized using the trimmed mean of *M*-values (TMM) method (Robinson and Oshlack, 2010). Where expression data from previous MORPH releases was used, the original clustering solutions were used as well. Gene expression data sets that were not included in previous MORPH analyses (*M. truncatula, P. trichocarpa*, and *Z. marina*) were clustered using CLICK (Sharan and Shamir, 2000). For *M. truncatula*, a metabolic clustering, with pathways as clusters, was included as well.

### MORPH bulk runs

To efficiently apply MORPH in a genome wide fashion, a Python3.5 command line interface (CLI) was developed named “morph-bulk.” The morph-bulk CLI uses the highly computationally efficient MORPH C++ implementation (v1.0.6) enabling very fast genome wide MORPH analyses. The morph-bulk CLI enables performing MORPH bulk runs in automatic pipeline mode or step by step, allowing full control over the analysis pipeline. The morph-bulk CLI, including installation instructions and a step-by-step protocol for MORPH bulk analyses, is available at https://github.com/arzwa/morph-bulk. We also provide a Singularity container (Kurtzer et al., 2017) further ensuring portability of the software.

The analysis pipeline proceeds as follows: first, a new species is automatically “added” to MORPH by generating the required configuration files based on the input data (expression matrices and corresponding clustering solutions). The different MORPH jobs are then defined for a given functional annotation (e.g., GO or MapMan) by taking the sets of genes annotated with a specific ontology term for example, and using them as input bait genes. MORPH is then run in bulk on all bait gene sets. Jobs with fewer than 5 genes in all data sets are discarded since co-expression based GBA methods are expected to give unreliable results for few genes, especially in the module partitioned framework used by MORPH. If desired, random MORPH bulk runs can then be performed to perform permutation test based significance assessment. In a random run, for each desired bait set size, *n* random sets of bait genes are picked from a randomly chosen data set and used in MORPH. The applied range of bait set sizes was from 5 to 30 and the number of random bait sets to analyze for each bait set size was set at 1,000. The corresponding AUSR values are recorded and used to empirically estimate the probability to observe AUSR value for a gene set size. This *p*-value for a bait gene set of size *S* with observed AUSR^*^ is then defined as the fraction of occurrences of AUSR scores larger than AUSR^*^ among 1,000 random gene sets of size *S*. The empirical probability distributions constructed in this fashion are shown in Figure S1. We note that no considerable differences were observed when using random gene sets drawn from the pool of functionally annotated genes vs. random sets drawn from the full genome (Figure S2). For extended annotation purposes, these *p*-values were corrected for multiple testing using the Benjamini and Hochberg procedure (Benjamini and Hochberg, 1995). After the main analysis, the results are summarized and extended functional annotations are generated. If desired, a Resource Description Framework (RDF) graph for MorphDB can be generated using the same CLI.

### The MorphDB database and web tool

MORPH bulk run data was parsed into an RDF graph using the *rdflib* (v4.2.2) Python package. The main objects (subjects) in the RDF graph are genes, gene sets (GO/MapMan terms), gene families and scores of a gene for a specific gene set term. The full list of predicates and objects is included in the about section of the MorphDB website. The RDF graph as constructed using *rdflib* was serialized to Turtle format [W3C recommendation (W3C, 2014)] and loaded in a triple store using Apache Jena (The Apache Software Foundation, 2011), queryable with the SPARQL query language (W3C, 2008). For in-browser network construction and visualization, the Cytoscape.js JavaScript library was used (Franz et al., 2015).

## Results

### MORPH bulk mode

The MORPH algorithm for candidate gene prediction uses as input a set of genes known to belong to a specific pathway or to have a common function (this set is referred to as the *bait* set) and aims to propose and rank additional genes of the same function or pathway. Expression profiles, their clustering solutions, and biological networks are used in the prediction. We applied MOPRH in a genome-wide fashion, hereafter called MORPH bulk mode, on six important model organisms: *Arabidopsis thaliana, Medicago truncatula, Solanum lycopersicum, Solanum tuberosum, Oryza sativa*, and *Populus trichocarpa*, and two non-model organisms, the recently sequenced seagrass *Zostera marina* (Olsen et al., 2016) and the medicinal plant *Catharanthus roseus*. In genome-wide runs, we provide as input to MORPH a genome-scale functional annotation as acquired from public repositories or popular software tools (e.g., Blast2GO or InterProScan). As bait gene sets, Gene Ontology (GO) annotations (Ashburner et al., 2000) were used, as well as MapMan annotations (Thimm et al., 2004) when available. MORPH uses a machine learning approach for performance estimation based on LOOCV and reports the area under the self-rank curve (AUSR) as a metric for the performance on a specific bait gene set. The AUSR ranges from 0 to 1 (perfect score), but its reliability is strongly dependent on the size of the bait gene set. Smaller sets are more likely to have larger AUSR values by chance. Therefore, for each bait gene set analyzed with MORPH, empirical *p*-values were computed using a permutation test. For each candidate, MORPH calculates a within-module Pearson correlation co-expression metric and subsequently converts these values to *z*-scores, which enables a common ranking across different modules. This *z*-score can then be used to rank and select relevant candidates.

Investigating the overall performance of MORPH illustrates its potential for gene function prioritization. Table 1 shows the number of significant bait sets for different *p*-value thresholds. MORPH performed best for *A. thaliana*, with 1,985 (66%) of the 3,005 GO terms and 279 (64%) of the 467 MapMan categories showing significant AUSR scores (*p* < 0.05). For *M. truncatula, O. sativa* and *P. trichocarpa*, a considerably smaller fraction of the analyzed gene sets showed significant AUSR scores (43, 33, and 24% respectively). For the Solanaceae species included (*S. lycopersicum* and *S. tuberosum*), fewer bait sets had good scores, probably due to a more limited GO annotation.

**Table 1:** Number of bait gene sets (GO/MapMan) successfully analyzed with MORPH in bulk mode.

		***A. thaliana***	***M. truncatula***	***P. trichocarpa***	***O. sativa***	***S. lycopersicum***	***S. tuberosum***	***C. roseus***	***Z. marina***
GO	# gene sets	3,005	2,461	2,903	2,528	1,232	1,540	907	1,272
	*p* < 0.10	2,182	1,303	947	1,054	854	1,207	650	1,028
	*p* < 0.05	1,985	1,046	711	846	736	1,099	591	893
MapMan	# gene sets	467	488	461	367	170	–	–	–
	*p* < 0.10	312	299	150	152	125	–	–	–
	*p* < 0.05	279	251	111	116	111	–	–	–

*Only bait gene sets with 5 or more genes in an expression data set are analyzed. p-values were calculated based on the empirical probability distribution of AUSR values for the relevant gene set size in the relevant species*.

**Figure 1:**
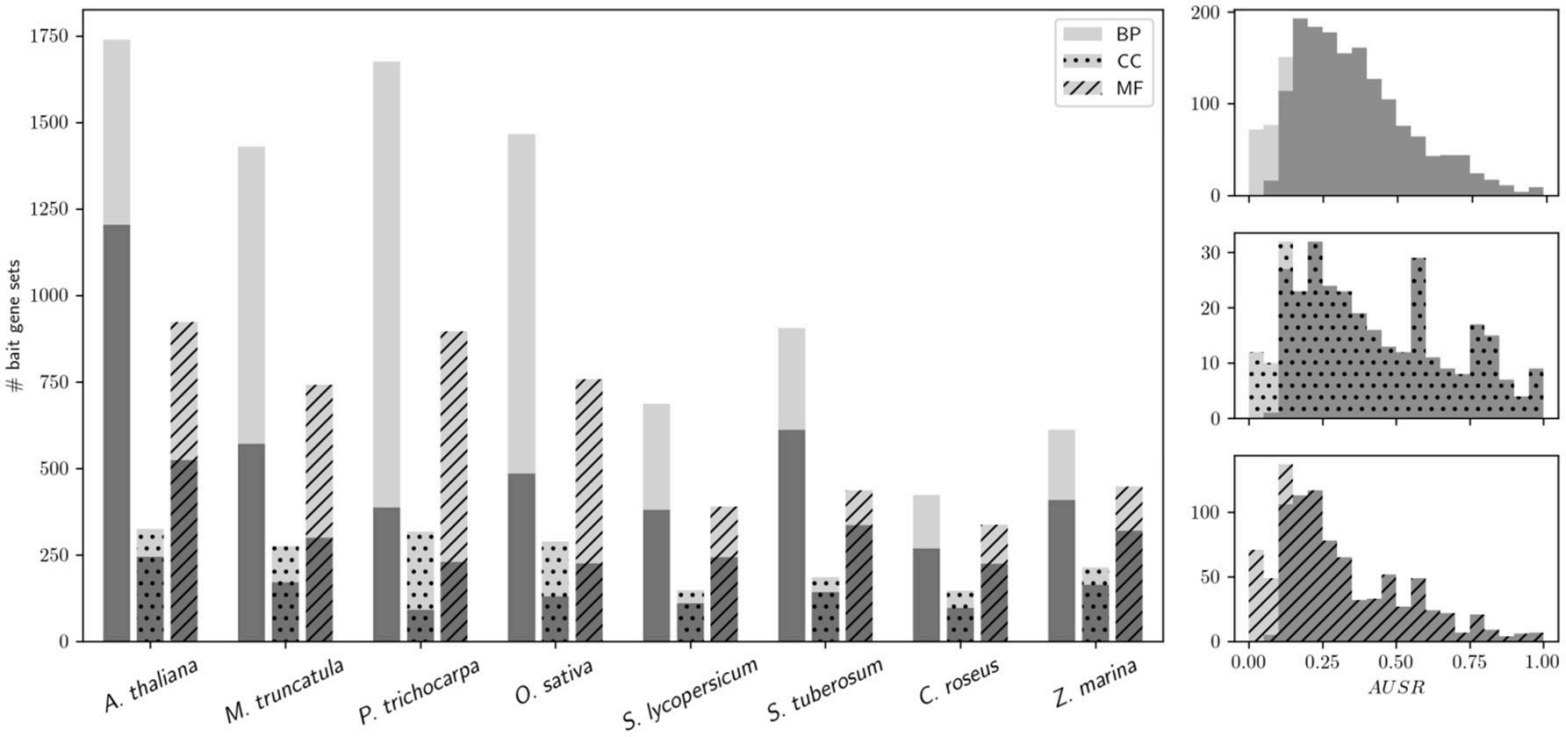
Performance of MORPH in bulk mode for different GO sub-ontologies. **(Left)** MORPH bulk results partitioned by sub-ontology (BP, CC, and MF) for the different species under study are shown. Light gray: the total number of analyzed bait gene sets with MORPH; dark gray: the number of significantly scoring (*p* < 0.05) bait gene sets. **(Right)** Histograms of AUSR values for different sub-ontologies in *A. thaliana* (using the same colors as in the left plot).

The performance of MORPH strongly depends on the available functional annotation, the expression data, and the clustering solutions. Interestingly, performance seems not to differ dramatically among different GO sub-ontologies, namely Biological Process (BP), Cellular Compartment (CC) and Molecular Function (MF), as shown in Figure 1, indicating that module-partitioned co-expression is manifest in every sub-ontology. However, a closer look reveals that, with the exception of *O. sativa* and *P. trichocarpa*, CC categories seem to systematically have higher fractions of significantly scoring gene sets. This observation may be explained by the fact that in all species the average gene set size is larger for CC GO categories than for BP or MF categories, e.g., 76 (CC) compared to 53 (BP) and 58 (MF) for *A. thaliana*, or 64 (CC) compared to 25 (BP) and 26 (MF) for *M. truncatula*. For larger gene sets, the AUSR may be significant even when the underlying co-expression strength is moderate. This can occur when a large set of bait genes that shows moderate overall co-expression contains some strongly co-expressed clusters of genes, which is a scenario that is directly addressed by the MORPH algorithm. While the BP ontology is probably the most directly relevant for candidate gene prediction, the other ontologies are also informative and hence included in MorphDB.

### Extending MORPH to non-model organisms

We used MORPH in bulk mode to predict putative gene functions for two non-model organisms, namely *C. roseus* and *Z. marina*. As in-depth functional annotations are not available for these organisms, we used established sequence-based algorithms to obtain predicted GO terms. This is common practice when analyzing newly sequenced organisms and non-model organisms (Amar et al., 2014). Using the predicted GO terms as bait sets, we applied MORPH using 5% FDR-corrected *p*-values for determining sets with significant predictions. For significant GO terms, we selected the top genes whose co-expression *z*-score was larger than the 97.5% percentile of the theoretical null distribution (*z* > 1.96). Our analysis resulted in 521 GO terms that could be assigned to 18,842 genes for *C. roseus*. Of these genes, 11,738 are currently unannotated, resulting in a considerable improvement of the primary GO annotation for these specific *z*-score cut-offs. For *Z. marina*, 794 GO categories were significant, which could be assigned to 13,343 genes using the same procedure as for *C. roseus*. Of these, 3,060 genes had no GO terms assigned previously, again showing the potential of this approach for improving automatically generated GO annotations with putative gene functions.

The analysis above may result in a high false positive rate and the resulting functional predictions are to be taken as a set of possible annotations that should be further tested. Nevertheless, we here show some specific examples of how these results can be used for generating biological hypotheses for *C. roseus*, for which the community is particularly interested in specialized metabolism pathways. *C. roseus* is an important medicinal plant that serves as a source for the potent indole alkaloid chemotherapeutic compounds vinblastine and vincristine (Almagro et al., 2015). Mining basic functional annotation data will often not suffice for finding interesting unknown regulators and pathway genes, while constructing co-expression networks and analyzing them can become very laborious and complicated. Mining functional annotations extended by MORPH offers an alternative. For example, considering transcription factors that are assigned by MORPH to aromatic compound biosynthetic processes (GO:0019438 and similar categories), several interesting candidates are suggested. Three top-scoring candidates (*z* > 3.0) are shown in Table 2. These genes also had high scores for other relevant GO terms, such as flavonoid and quercetin (also a flavonoid) metabolism related terms as well as response to wounding and other more general metabolism related terms (O-methyltransferase, NAD binding, Thiamine pyrophosphate (TPP) binding and malate metabolism). Flavonoids, as well as other phenylpropanoid compounds, are well known for their roles in plant defense (Falcone Ferreyra et al., 2012; Tohge et al., 2013). Plant defense responses are well known to correlate with enhanced production of many specialized metabolites, and such responses have been described for *C. roseus* as well (Menke et al., 1999; Roepke et al., 2010). This simple example demonstrates how extended functional annotations acquired by MORPH can provide a valuable starting point for identifying interesting candidates for pathways of interest in non-model genomes.

**Table 2:** Subset of transcription factors in *C. roseus* associated with GO:0019438 (aromatic compound biosynthetic process) by MORPH and their respective other predicted GO terms.

**Gene**	**Description**	**GO Term**
*Caros015806.1*	ethylene-responsive transcription factor 1B-like	GO:0019438, aromatic compound biosynthetic process
		GO:0004471, malate dehydrogenase (decarboxylating) activity
		GO:0008171, O-methyltransferase activity
		GO:0006108, malate metabolic process
		GO:0009611, response to wounding
		GO:0051287, NAD binding
*Caros031076.1*	AP2/ERF domain-containing transcription factor	GO:0019438, aromatic compound biosynthetic process
		GO:0008171, O-methyltransferase activity
		GO:0080044, quercetin 7-O-glucosyltransferase activity
		GO:0080043, quercetin 3-O-glucosyltransferase activity
		GO:0052696, flavonoid glucuronidation
		GO:0009813, flavonoid biosynthetic process
*Caros003741.1*	WRKY transcription factor	GO:0019438, aromatic compound biosynthetic process
		GO:0030976, thiamine pyrophosphate binding
		GO:0008171, O-methyltransferase activity
		GO:0052696, flavonoid glucuronidation

*Only bait gene sets with an AUSR score with corresponding adjusted p-value < 0.05 are included and only their candidate genes with z > 1.96 are annotated with the GO category under consideration*.

### MorphDB

**Figure 2:**
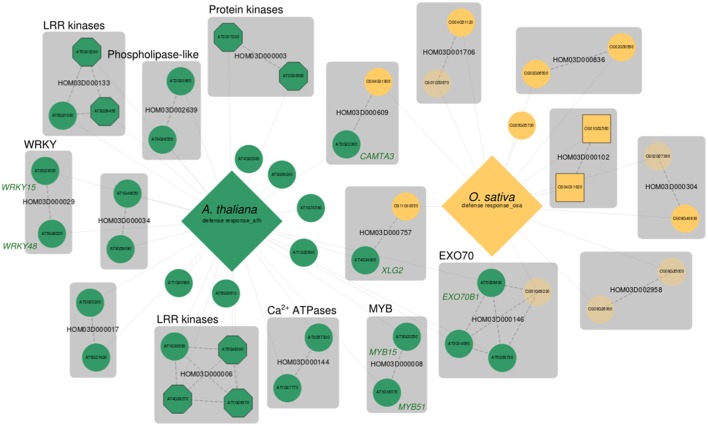
Comparative MorphDB network of GO:0006952 (defense response) for *A. thaliana* and *O. sativa. A. thaliana* genes are indicated in green, *O. sativa* genes indicated in yellow. Only genes among the top 100 candidates for this process with a homologous relation to another candidate in MorphDB for the same GO category are included for clarity. Large diamonds represent the bait gene set and candidate genes are connected to their respective bait set. Bait genes themselves are omitted for clarity as well. Genes with no functional description are depicted transparently. Transporters are shown as squares and kinases and receptors as octagons. All other candidate genes are shown as circles. Gene families are shown as gray compound nodes and orthology relationships as dashed edges.

We created a web-based tool named MorphDB that provides access to MORPH's predictions for the six important model organisms discussed above, as well as for *C. roseus* and *Z. marina*. For each species, MorphDB stores the top 100 candidates with *z*-scores that exceed the 90% percentile of the theoretical null distribution (*z* > 1.28) for all gene sets with empirical *p*-value < 0.10. The primary goal of MorphDB is to integrate the MORPH candidate gene predictions across species using orthogroup data as retrieved from the PLAZA comparative genomics platform (Proost et al., 2009; Van Bel et al., 2012). Both candidates and bait genes are linked to their respective homologous candidates and to bait genes in the other species in the MorphDB database. MorphDB allows querying in a gene centric manner, enabling users to provide a set of genes of interest and view the GO categories or MapMan terms that were predicted for it by MORPH. Moreover, gene sets can also be queried and visualized in a comparative network, i.e., across species, which allows identification of candidate genes that manifest conserved signatures across different species (e.g., Figure 2). This analysis can be used for highlighting candidates in less thoroughly studied species based on knowledge in other organisms. Lastly, MorphDB has a SPARQL endpoint, allowing arbitrarily complex queries of the database. We illustrate the use and the potential of the tools in MorphDB in the next section.

### Prioritizing regulatory genes for the plant defense response

In this section, we focus on plant defense responses and related specialized metabolism pathways in *A. thaliana, M. truncatula, O. sativa, S. tuberosum*, and *S. lycopersicum*. A case study is shown as an illustration of the potential of MorphDB.

Figure 2 shows a comparative network generated by MorphDB for the GO category “defense response” (GO:0006952) in *A. thaliana* (*AUSR* = 0.15, *p* = 0.02, 269 bait genes), and *O. sativa* (*AUSR* = 0.23*, p* = 0.02, 150 bait genes). The network shows mainly signaling related genes for *A. thaliana*, with high-scoring gene families such as HOM03D000133 and HOM03D000006 (Leucine-rich receptor (LRR) kinases, all *z* > *2*.42), HOM03D000003 (protein kinases, all *z* > 2.76), HOM03D002639 (phospolipase-like proteins, both *z* > 2.50) and HOM03D000144 (autoinhibited Ca^2+^ ATPases, both *z* > 2.90). Besides these putatively signaling related genes, putative transcription factors (TFs) are retrieved, such as WRKY TFs (HOM03D000029) and MYB domain TFs (HOM03D000008). Both *AT2G23320* (WRKY15, *z* = 2.71) and *AT5G49520* (WRKY48, *z* = 2.40) have been associated with the response to chitin, an import plant-defense elicitor from fungal origin (Libault et al., 2007). WRKY48 was also shown to be involved in the defense response to bacterial pathogens (Xing et al., 2008). Both TFs have been associated with diverse stress responses in another large scale computational study (Heyndrickx and Vandepoele, 2012). Both *AT3G23250* (MYB15, *z* = 2.44) and *AT1G18570* (MYB51, *z* = 2.42) have been associated with a whole range of hormone metabolism and stress response related processes. MYB51 regulates glucosinolate biosynthesis (Gigolashvili et al., 2007; Frerigmann et al., 2012), specialized metabolites that act as antiherbivore compounds in plants. MYB15 has been associated with the response to chitin (Libault et al., 2007).

In addition, a highly remarkable group of predicted candidates from the HOM03D000146 gene family is retrieved. These genes belong to the EXO70 gene family, which are putative exocyst subunits conserved in land plants (Chong et al., 2010; Wang et al., 2010). EXO70B1 has been associated with autophagy-related transport in *A. thaliana* (Kulich et al., 2013), a crucial process in diverse plant stress responses. Interestingly, Zhao et al. (2015) reported that *exo70B1* mutants showed enhanced defense response through activation of a nucleotide binding domain and leucine-rich repeat-containing (NLR)-like disease resistance protein. Their study provides a link between the plant immune system and the exocyst complex, and they suggest that pathogen effectors may manipulate and interact with the plant secretion machinery. The MORPH results presented here support this hypothesis, as for two species, independently, exocyst related proteins are among the top 100 candidates with acceptable to high scores (*AT5G58430 (EXO70B1)*: *z* = 2.58, *AT3G14090 (EXO70D3)*: z = 2.57, *AT5G59730 (EXO70H7)*: *z* = 2.69 and *OS01G69230*: *z* = 1.44). Looking at the processes for which *EXO70B1* was predicted as a candidate in *A. thaliana* (Table 3), several defense related GO terms are obtained (e.g., GO:0010337, GO:0031347, GO:0009410, GO:0009816, and GO:0008219), further supporting the hypothesis of exocyst related functions in plant defense responses.

**Table 3:** GO terms for which *AT5G58430* is among the top 100 MORPH-predicted candidates.

**GO term**	**Term description**	**# bait genes**	**AUSR**	***p*-value**	**z-score**
GO:0009612	Response to mechanical stimulus	53	0.58	0.00	2.80
GO:0010337	Regulation of salicylic acid metabolic process	7	0.48	0.00	2.84
GO:0031347	Regulation of defense response	100	0.42	0.00	2.62
GO:0043623	Cellular protein complex assembly	25	0.42	0.00	2.33
GO:0052541	Plant-type cell wall cellulose metabolic process	25	0.38	0.00	2.46
GO:0004805	Trehalose-phosphatase activity	13	0.35	0.00	2.51
GO:0046351	Disaccharide biosynthetic process	14	0.32	0.00	2.28
GO:0001871	Pattern binding	15	0.30	0.00	2.56
GO:0009410	Response to xenobiotic stimulus	45	0.29	0.00	2.30
GO:0005484	SNAP receptor activity	19	0.26	0.00	2.72
GO:0009816	Defense response to bacterium, incompatible interaction	43	0.23	0.00	3.15
GO:0009652	Thigmotropism	5	0.51	0.01	2.52
GO:0009312	Oligosaccharide biosynthetic process	15	0.27	0.01	2.48
GO:0005991	Trehalose metabolic process	16	0.26	0.01	2.28
GO:0008219	Cell death	43	0.16	0.01	2.13
GO:0006952	Defense response	269	0.15	0.02	2.58
GO:0009690	Cytokinin metabolic process	21	0.18	0.03	2.14

*Only GO terms with an AUSR score corresponding to p < 0.05 are shown*.

Other candidate gene predictions that are consistent over species can be retrieved, such as the candidates found in gene families HOM03D000609 and HOM03D000757. The first family again consists of an already known defense response regulator in *A. thaliana*, namely *AT2G22300* (*z* = 2.48) encoding CAMTA3 (Calmodulin-binding transcription activator), a putative CAM binding TF. *CAMTA3* mutants (*camta3-1* and *camta3-2*) show enhanced defense responses, with a high fraction of defense associated upregulated genes in both the *camta3-1* and *camta3-2* mutant (Galon et al., 2008). The homolog in *O. sativa* (*OS04G31900*) predicted by MORPH for GO:000652 has not been associated with defense responses before. Lastly, HOM03D000757 also has a candidate predicted in both *A. thaliana* (*AT4G34390, z* = 2.82) and *O. sativa* (OS11G10050*, z* = 1.44). *AT4G34390* encodes an extra-large GTP-binding protein (XLG2), which has been shown to be involved in root morphogenesis (Ding et al., 2007) and defense responses to bacteria (Zhu et al., 2009). Again, as expected, the rice homolog predicted by MORPH has not been functionally characterized, and the MORPH prediction supports the hypothesis of a conserved function in defense responses.

**Figure 3:**
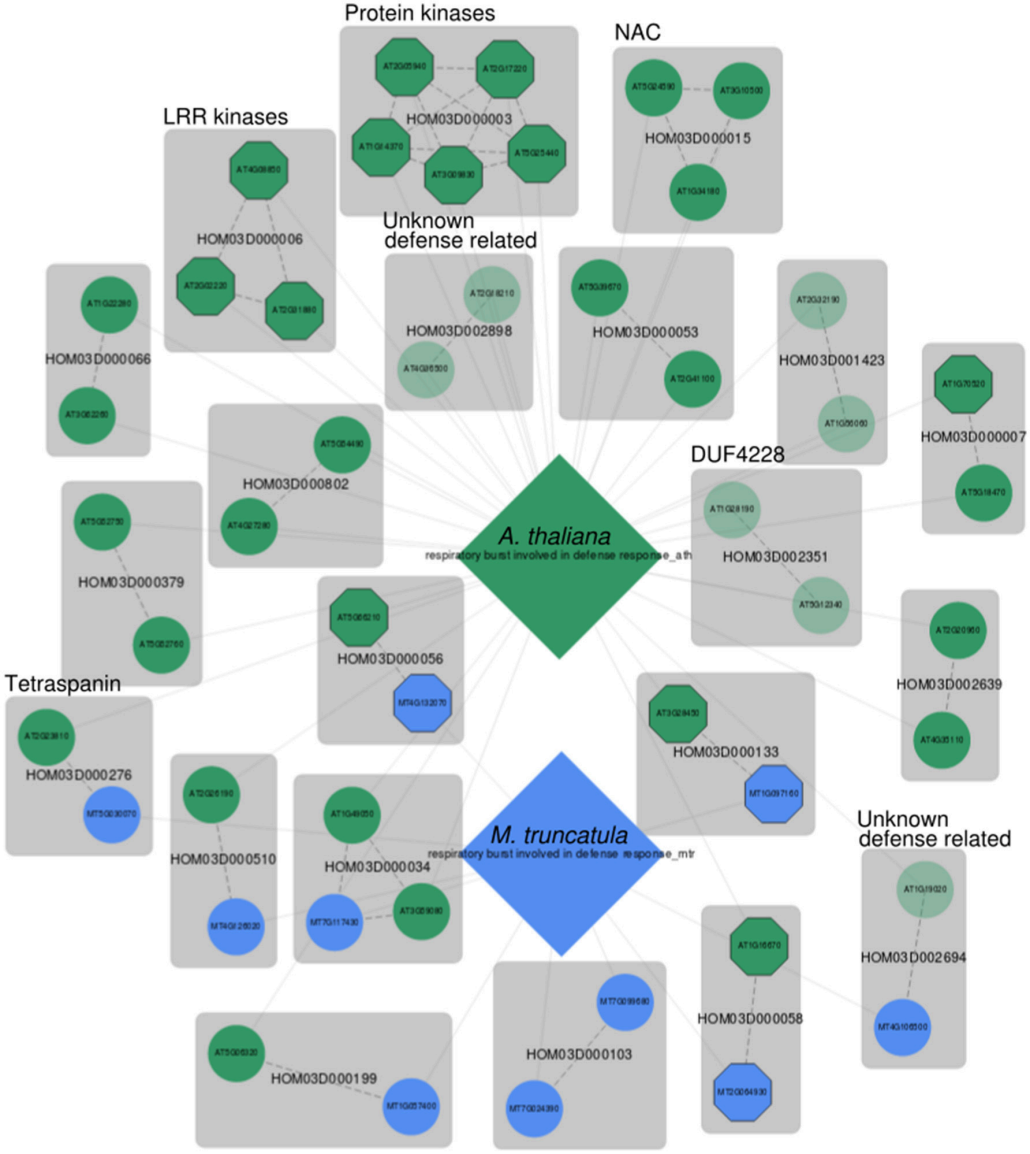
MorphDB network for GO:0002679 (respiratory burst involved in defense response) for *A. thaliana* (green) and *M. truncatula* (blue). For more details, see Figure 2.

A more specific defense response related GO term that is also well represented in MorphDB is GO:0002679 (respiratory burst involved in defense response). The respiratory burst is defined as the biological process in which elevated metabolic activity increases oxygen consumption, and through an NADH dependent system reactive oxygen species are formed (ROS), such as hydrogen peroxide (Kawano, 2003). Again, a MorphDB network was constructed, with a focus on comparative aspects between *A. thaliana* (AUSR = 0.74, *p* < 0.005) and *M. truncatula* (AUSR = 0.54, *p* < 0.01) (Figure 3). Below, we focus on several interesting observations that can be made from the network.

Interestingly, several unknown *Arabidopsis* genes are predicted as candidates for this biological process. For HOM03D002351, both *Arabidopsis* gene family members are among the top 100 MORPH predicted candidates. This gene family consists of proteins with a domain of unknown function (DUF) DUF4228, which is functionally uncharacterized. One of the two *Arabidopsis* duplicates (*AT1G28190*) has been linked to defense response related processes (JA and SA signaling and hypersensitive response) in a large-scale systems biology study (Heyndrickx and Vandepoele, 2012). The other *Arabidopsis* homolog (*AT5G12340*) has no functional term assigned and was predicted to have a mitochondrial subcellular localization, which is consistent with a putative role in respiratory burst. Interestingly, the unknown gene *AT5G12340* is ranked higher (*z* = 3.01) than the previously associated homolog *AT1G28190* (*z* = 2.71). An inspection of the phylogenetic tree of this gene family on PLAZA shows that the family is angiosperm (Magnoliophyta) specific and that it is conserved across this clade. The tree indicates that the duplication event from which the *Arabidopsis* homologs are derived precedes the divergence of the angiosperms, as inferred from the position of the *Amborella trichopoda* homologs in the tree. The ancient origin of this gene family and the conservation across the angiosperm tree indicates a high likelihood of functional importance.

HOM03D002694 is another gene family without functional characterization. The Arabidopsis gene *AT1G19020* has been shown to be involved in oxidative stress signaling in a mutant phenotype screen (Luhua et al., 2008), and has been associated with response to wounding, response to insect, SAR, SA mediated signaling and defense response to fungus by Heyndrickx and Vandepoele (2012). A gene centric search in MorphDB shows that *AT1G19020* is predicted for a plethora of stress and defense response related GO terms (Table S1). A similar gene centric search in MorphDB reveals that the functionally uncharacterized Medicago homolog *MT4G106500* is also predicted to be involved in anthocyanin-containing compound biosynthesis (GO:0009781). Anthocyanin biosynthesis is regulated by JA signaling (Shan et al., 2009) and anthocyanin accumulation is associated with enhanced herbivore resistance in *Arabidopsis* (Khan et al., 2016).

Interestingly, tetraspanin gene family members (HOM03D000276) are also present in the network for both *Arabidopsis* and *Medicago*. This family of membrane proteins has been mainly studied in the context of development (Wang et al., 2012, 2015) and it has been suggested that tetraspanins have a role in cell-cell communication during various developmental stages. However, it has been observed that many tetraspanins remain active also in mature differentiated tissues (Wang et al., 2015), and some tetraspanin promoter regions contain defense and pathogen response elements (Wang et al., 2015). Therefore, it is tempting to suggest a role in defense response through sensing of pathogen related molecules, because of the putative role in developmental cell-cell communication, the presence of extracellular loops and the presence of pathogen response related promoter elements. Also, this gene family has undergone several duplications and has been shown to contain putative functionally divergent clades (Wang et al., 2012), supporting the possibility of tetraspanins involved in defense response.

**Figure 4:**
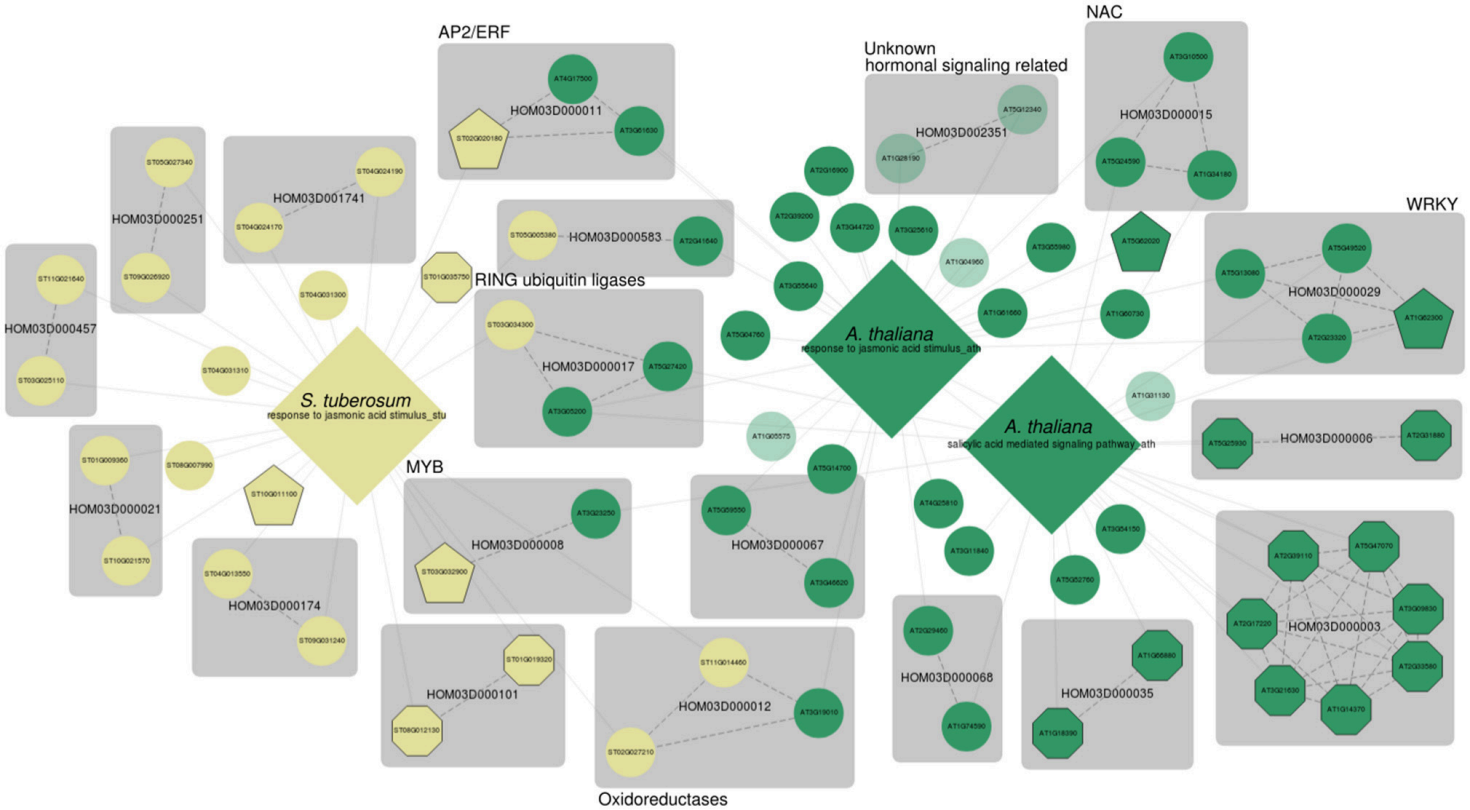
MorphDB network for GO:0009753 (response to jasmonic acid stimulus) and GO:0009863 (SA mediated signaling pathway) for *A. thaliana* (green) and *S. tuberosum* (pale yellow). Only the top 50 candidates for each GO category that have a homolog predicted in some other species in MorphDB are shown. For more details, see Figure 2.

Lastly, we analyzed jasmonate (JA) and salicylic acid (SA) signaling. SA is one of the major important signaling molecules involved in the plant defense response (Loake and Grant, 2007; Zhang et al., 2013). SA biosynthesis is activated in response to a wide variety of phytopathogens, and SA mediated signaling results in the accumulation of pathogenesis-related (PR) proteins (Loake and Grant, 2007). It is the main molecular signal involved in the establishment of both local and systemic acquired resistance (SAR) (Loake and Grant, 2007). Besides its roles in defense and disease resistance, SA is known to regulate leaf senescence, flowering and thermogenesis (Dempsey et al., 2011; Zhang et al., 2013). Next to salicylic acid (SA) mediated signaling, JA mediated signaling is the main signaling pathway for plant defense responses (Turner et al., 2002; Chini et al., 2007), and JA is thought to be the key regulator for many specialized metabolism pathways that are triggered during biotic and abiotic stresses. Investigation of functional gene sets for SA and JA mediated signaling is therefore highly relevant in the context of this case study. MORPH results for GO:0009753 (response to jasmonic acid stimulus) for *A. thaliana* (AUSR = 0.27, *p* < 0.01), and *S. tuberosum* (AUSR = 0.26, *p* < 0.01) were based on gene sets of 236 and 74 bait genes respectively. GO:0009863 (SA mediated signaling pathway) scored an AUSR of 0.44 (*p* < 0.001) for a bait set consisting of 132 genes. The network of the top 50 candidates is shown in Figure 4.

Again, the results illustrate the strength of a systematic network-based analysis. Many relevant gene families are identified, some with putative TF genes. For example, the MYB TF family HOM03D00008, which has roles in defense related specialized metabolism (Liu et al., 2015), is represented in the network. The gene family HOM03D000011 consists of AP2/ERF domain containing TFs, and the selected *Arabidopsis* gene *AT4G17500* in this family has been associated with defense response (Fujimoto et al., 2000; Onate-Sanchez and Singh, 2002). HOM03D000029 consists of WRKY domain containing TFs, already discussed above. HOM03D000015 consists of NAC DNA-binding domain containing proteins of which NAC16, NAC53 and TCV-interacting protein are among the top 50 candidates for either GO:0009751 or GO:0009863 in *A. thaliana*, all with high co-expression scores (*z* > 2.59). NAC proteins are widely recognized for their roles in hormonally controlled development (Aida et al., 1997; Xie et al., 2000) and biotic and abiotic stress responses (Lee et al., 2012; Nuruzzaman et al., 2013) (NAC53). However, *A. thaliana* consists of 92 NAC domain containing proteins, of which many have not been functionally characterized in detail. NAC16 has been previously associated with the response to chitin in *Arabidopsis* (Libault et al., 2007) and TCV-interacting protein has been shown to physically interact with turnip crinkle virus (TCV) viral capsids (Ren et al., 2000; Donze et al., 2014).

Interestingly, a family of RING type ubiquitin ligases is also present in the network (HOM03D000017). Both *AT5G27420*, which encodes CNI1 (Carbon/Nitrogen Insensitive1, also known as ATL31), and *AT3G05200* (ATL6) have been linked previously to both fungal (Libault et al., 2007) and bacterial defense responses (Maekawa et al., 2012). The potato homolog *ST03G034300* has not been functionally characterized before, and MORPH strongly suggests a role in defense responses. An interesting family of oxidoreductases is obtained (HOM03D000012) as well, with putative functions in flavonoid biosynthesis. Lastly, this network also shows several totally uncharacterized genes, of which the *Arabidopsis* genes in the gene family HOM03D002351 seem particularly interesting. *AT1G28190* was previously associated with various hormonal signaling pathways, among which JA, SA, abscisic acid and ethylene signaling, by Heyndrickx and Vandepoele (2012) consistent with a putative role in defense. The homolog *AT5G12340* could only be associated with a mitochondrial subcellular localization and is further not functionally characterized. A gene centric query for *AT5G12340* shows that this gene is predicted as a high scoring candidate for a plethora of stress response related processes, further supporting a role in stress and defense responses. MORPH results as integrated in MorphDB can provide useful hints on gene functions for these enigmatic genes.

## Discussion

MORPH is a highly valuable tool that was developed to accelerate gene discovery for plant metabolic pathways (Tzfadia et al., 2012). Here, the usage of MORPH was reconsidered from a genome-wide and comparative viewpoint in the context of functional annotation, gene discovery and candidate gene prioritization. Besides a framework and methodology for performing MORPH bulk runs, a database and web-tool, MorphDB, were developed, providing a friendly interface for consulting MORPH bulk predictions of several important model organisms. An additional key feature of the MORPH bulk framework is the easy usage of MORPH with custom data or novel species, which was not supported previously. This enables researchers to use the MORPH algorithm for candidate gene prioritization in their species of interest and tackle specific research questions.

In this work, we showed how MORPH can be used in bulk mode on non-model species, such as *C. roseus* and *Z. marina*, for rendering putative gene functions by analyzing bait gene sets defined by GO categories. MORPH bulk runs were also performed for already well-studied organisms, with gene discovery and candidate gene prioritization as main objectives. The integration of MORPH results with homology information from PLAZA (Van Bel et al., 2012) in MorphDB, as well as the comparative network visualization implemented in the same web tool, were shown to be particularly useful for gene discovery and candidate gene prioritization objectives in a case study concerning the plant defense response in several model organisms. MORPH predictions were shown to be well in accordance with the literature or with previously described functions for homologous genes. Our findings illustrate the relevance and potential of MORPH predictions, which may be particularly interesting for the elucidation and prioritization of regulatory roles for members of large gene families of TFs and signaling genes. Indeed, while sequence similarity and profile based methods can easily assign a TF, kinase or receptor function based on characteristic protein domains, it remains virtually impossible to associate these functions with specific biological processes. For these purposes, gene expression based methods provide a valuable solution, as we have shown in our defense response case study.

In our case study, we showed how the integration of MORPH results with homology data strengthens hypotheses suggested by MORPH and renders highly interesting candidates. A key advantage of using MORPH in a comparative fashion over classical comparative co-expression networks such as in CoExpNetViz (Tzfadia et al., 2016) is that using candidate genes predicted by MORPH instead of the top co-expressed genes based on Pearson correlation values is expected to render a higher fraction of relevant candidates. Even relatively small MORPH networks can therefore render highly relevant candidates, with the additional advantage that these networks are relatively simple and easy to browse using the MorphDB resource. Here also the tight integration with the PLAZA platform accelerates biological discovery. The case study also showed that the visual highlighting of classes of regulatory sets of genes (defined here as: transcription factors, kinases, receptors and transporters) is quite helpful in browsing the networks for interesting candidates efficiently. As functional biology is shifting to multi-omics analyses, interest in network based approaches for visualization and data exploration is growing. Networks enable the easy integration of additional experimental data such as proteomic, protein-protein interaction or genetic interaction data.

We expect that gene expression based analysis will remain central in the future elucidation of gene functions. High performing candidate gene prioritization algorithms such as MORPH enable further in-depth exploration of the functional gene space in both model and non-model organisms, but the results remain largely speculative. This is in contrast with similarity based approaches, which render quite confident gene functions but result in a largely incomplete exploration of the functional landscape of a genome. Applying MORPH in bulk mode enables researchers to generate a large set of putative functional associations, which can be further mined by domain experts to address specific research questions, as we have shown in this work. Moreover, the MorphDB web resource enables efficient querying and interpretation in a comparative setting, further aiding researchers in the prioritization of candidate genes for their particular biological process of interest.

## Author contributions

AZ, OT, and YV designed the research; AZ and TD developed MORPH and MORPH bulk; AZ and TV developed the MorphDB database and web interface; AZ, OT, YV, DA, and RS analyzed data and wrote the manuscript.

### Conflict of interest statement

The authors declare that the research was conducted in the absence of any commercial or financial relationships that could be construed as a potential conflict of interest.
