# Supplementary material for: MorphDB: Prioritizing Genes for Specialized Metabolism Pathways and Gene Ontology Categories in Plants
Source: Front Plant Sci. 2018 Mar 19;9:352. doi: 10.3389/fpls.2018.00352 (PMC5867296; doi:10.3389/fpls.2018.00352)
Supplement: Supplementary file 1 [file Table1.DOCX]

Supplementary Material

MorphDB: Prioritizing Genes for Specialized Metabolism Pathways and Gene Ontology Categories in Plants

Arthur Zwaenepoel^1,2,3^, Tim Diels^1,2,3^, David Amar^4^, Thomas Van Parys^1,2,3^, Ron Shamir^5,^ Yves Van de Peer^1,2,3,5*^ & Oren Tzfadia^1,2,3*^

^1^Department of Plant Biotechnology and Bioinformatics, Ghent University, 9052 Ghent, Belgium

^2^VIB Center for Plant Systems Biology, 9052 Ghent, Belgium

^3^Bioinformatics Institute Ghent, Ghent University, 9052 Ghent, Belgium

^4^Stanford Center for Inherited Cardiovascular Disease, Stanford University, Stanford, CA 94305, USA

^5^Blavatnik School of Computer Science, Tel-Aviv University, Tel-Aviv, Israel.

^6^Genomics Research Institute, University of Pretoria, Pretoria 0028, South Africa

*** Correspondence:
Oren Tzfadia**
**ortzf@psb.vib-ugent.be; yvpee@psb.ugent.be**

# Supplementary Figures and Tables

## Supplementary Figures


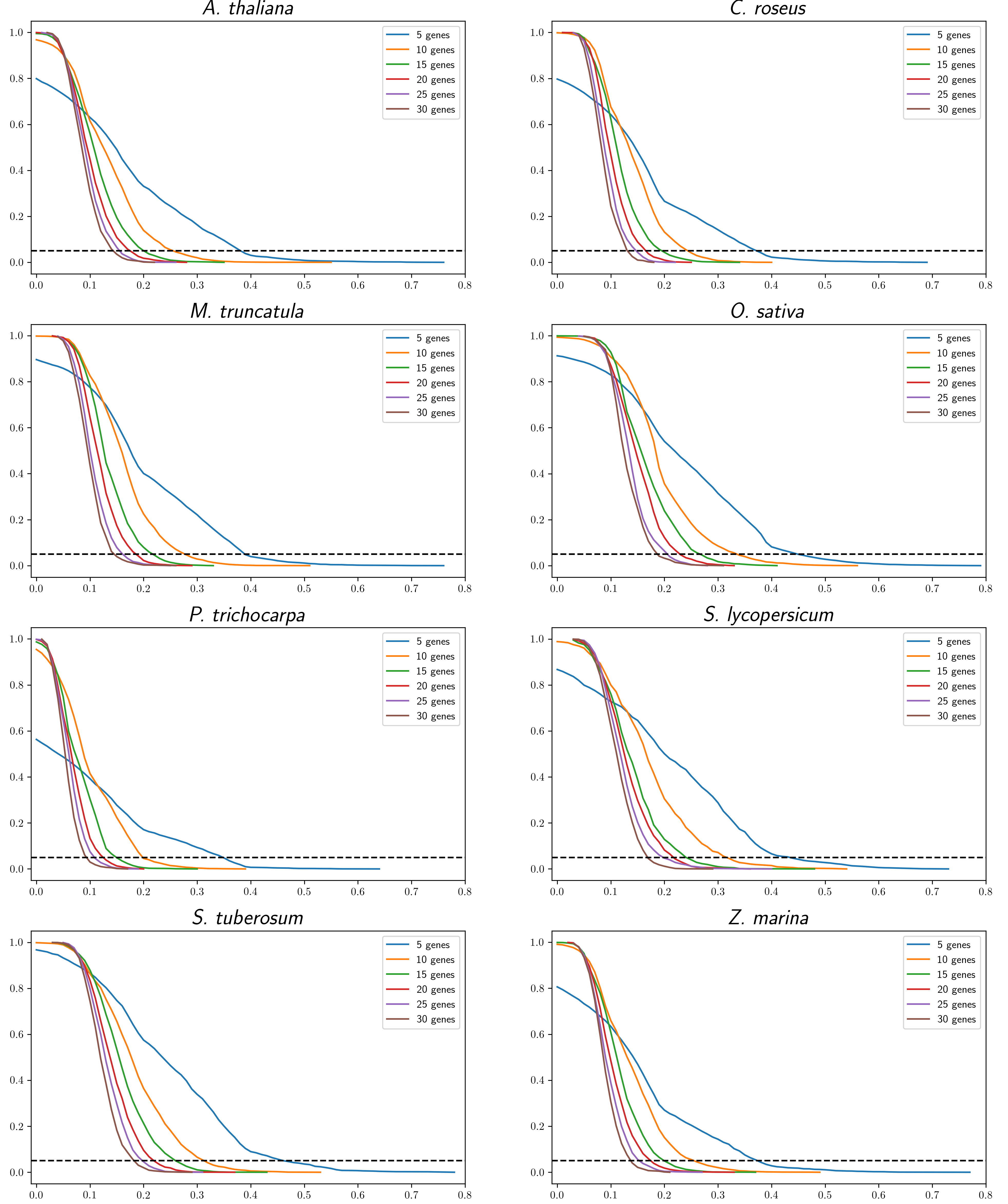


**Supplementary Figure** 1**.** **Empirical probability distribution of AUSR scores for different bait gene set sizes.** The probability to observe an AUSR value among 1000 random bait gene sets of a particular size is plotted. The dashed line indicates the p = 0.05 level.


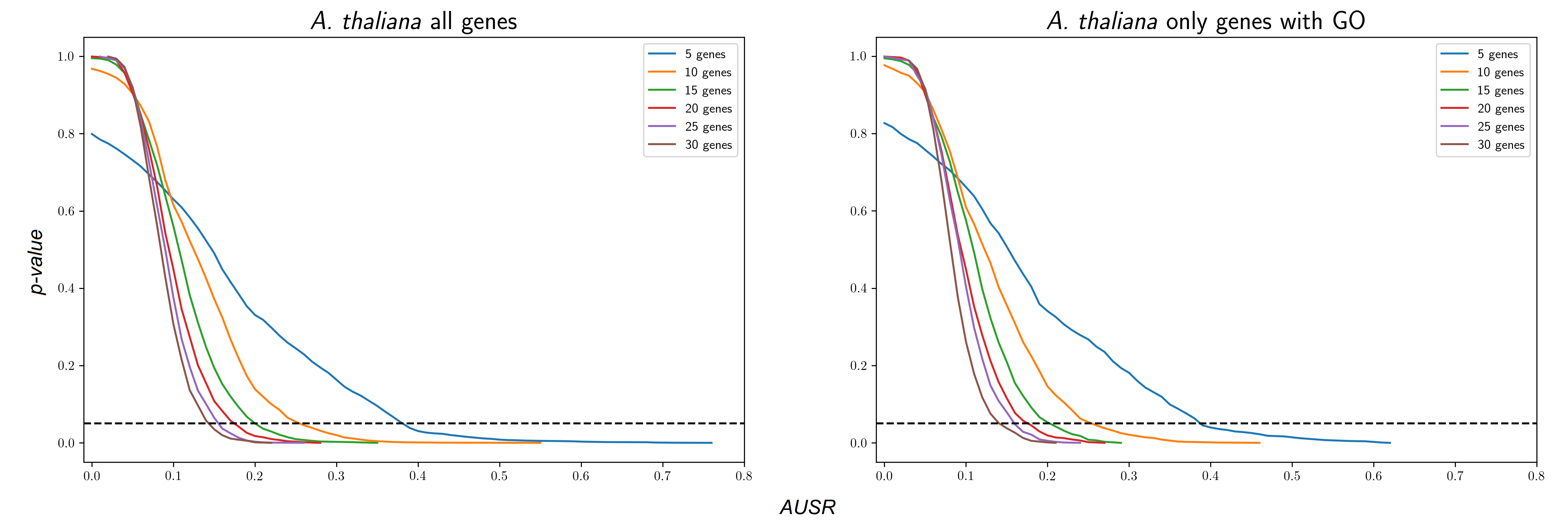


**Supplementary Figure** 2**. Empirical probability distributions for different background gene pools from which random bait sets are drawn.** Left: empirical probability distributions for different bait gene sets drawn from the full *A. thalian*a genome. Right: empirical probability distributions using random bait gene sets drawn from all *A. thaliana* genes with a GO annotation

# Supplementary Tables

**Supplementary Table 1. GO biological process terms with *p* < 0.01 for which *AT1G19020* is among the top 100 candidates.** The table below can be acquired using the gene centric query view on MorphDB.

| **GO term** | **Term description** | **AUSR** | **Bait set size** | ***z-score*** |
| --- | --- | --- | --- | --- |
| GO_0009819 | drought recovery | 0.78 | 5 | 2.58 |
| GO_0002679 | respiratory burst involved in defense response | 0.74 | 95 | 3.42 |
| GO_0052542 | defense response by callose deposition | 0.66 | 35 | 3.44 |
| GO_0004430 | 1-phosphatidylinositol 4-kinase activity | 0.66 | 6 | 1.79 |
| GO_0004867 | serine-type endopeptidase inhibitor activity | 0.59 | 10 | 2.64 |
| GO_0009612 | response to mechanical stimulus | 0.58 | 53 | 3.39 |
| GO_0044448 | cell cortex part | 0.56 | 12 | 2.51 |
| GO_0046903 | secretion | 0.56 | 12 | 2.51 |
| GO_0006984 | ER-nucleus signaling pathway | 0.53 | 10 | 2.48 |
| GO_0071456 | cellular response to hypoxia | 0.52 | 21 | 2.74 |
| GO_0009581 | detection of external stimulus | 0.48 | 17 | 3.00 |
| GO_0051865 | protein autoubiquitination | 0.47 | 10 | 3.23 |
| GO_0043069 | negative regulation of programmed cell death | 0.46 | 146 | 3.21 |
| GO_0009697 | salicylic acid biosynthetic process | 0.46 | 160 | 2.90 |
| GO_0009646 | response to absence of light | 0.46 | 30 | 3.23 |
| GO_0045087 | innate immune response | 0.46 | 83 | 2.82 |
| GO_0009863 | salicylic acid mediated signaling pathway | 0.44 | 132 | 3.00 |
| GO_0010286 | heat acclimation | 0.44 | 73 | 2.83 |
| GO_0009693 | ethylene biosynthetic process | 0.44 | 77 | 3.16 |
| GO_0015802 | basic amino acid transport | 0.43 | 27 | 2.50 |
| GO_0006862 | nucleotide transport | 0.42 | 26 | 2.45 |
| GO_0015696 | ammonium transport | 0.41 | 27 | 2.41 |
| GO_0042542 | response to hydrogen peroxide | 0.40 | 145 | 2.70 |
| GO_0052544 | defense response by callose deposition in cell wall | 0.40 | 29 | 2.73 |
| GO_0010583 | response to cyclopentenone | 0.39 | 109 | 2.80 |
| GO_0009061 | anaerobic respiration | 0.39 | 12 | 2.48 |
| GO_0009306 | protein secretion | 0.39 | 21 | 2.29 |
| GO_0006865 | amino acid transport | 0.37 | 117 | 2.50 |
| GO_0000165 | MAPK cascade | 0.35 | 174 | 2.82 |
| GO_0043090 | amino acid import | 0.34 | 70 | 2.53 |
| GO_0009695 | jasmonic acid biosynthetic process | 0.33 | 113 | 2.73 |
| GO_2000377 | regulation of reactive oxygen species metabolic process | 0.33 | 14 | 2.40 |
| GO_0000303 | response to superoxide | 0.33 | 30 | 2.27 |
| GO_0006612 | protein targeting to membrane | 0.33 | 327 | 2.95 |
| GO_0010363 | regulation of plant-type hypersensitive response | 0.33 | 327 | 2.94 |
| GO_0035556 | intracellular signal transduction | 0.32 | 207 | 3.10 |
| GO_0051707 | response to other organism | 0.32 | 58 | 2.68 |
| GO_0009620 | response to fungus | 0.32 | 92 | 3.06 |
| GO_0015085 | calcium ion transmembrane transporter activity | 0.31 | 14 | 2.36 |
| GO_0009867 | jasmonic acid mediated signaling pathway | 0.31 | 237 | 2.89 |
| GO_0015824 | proline transport | 0.31 | 56 | 3.16 |
| GO_0009738 | abscisic acid mediated signaling pathway | 0.28 | 205 | 2.88 |
| GO_0009753 | response to jasmonic acid stimulus | 0.27 | 236 | 2.64 |
| GO_0009626 | plant-type hypersensitive response | 0.25 | 51 | 2.53 |
| GO_0009266 | response to temperature stimulus | 0.24 | 109 | 2.49 |
| GO_0042538 | hyperosmotic salinity response | 0.24 | 140 | 2.64 |
| GO_0009873 | ethylene mediated signaling pathway | 0.24 | 148 | 2.78 |
| GO_0009723 | response to ethylene stimulus | 0.23 | 207 | 2.92 |
| GO_0009816 | defense response to bacterium, incompatible interaction | 0.23 | 43 | 3.09 |
| GO_0009414 | response to water deprivation | 0.18 | 311 | 2.47 |
| GO_0010193 | response to ozone | 0.18 | 35 | 2.49 |
